# Taxonomic studies on the genus *Isotrema* (Aristolochiaceae) from China: II. *I. brevilimbum* (Aristolochiaceae), a new species from Guizhou, China

**DOI:** 10.3897/phytokeys.152.51760

**Published:** 2020-07-03

**Authors:** Jun Wang, Ji-Dong Ya, Cheng Liu, Guang Liu, Feng Cao, Jin-Shuang Ma, Xin-Xin Zhu

**Affiliations:** 1 College of Life Sciences, Xinyang Normal University, Xinyang, 464000, Henan, China Xinyang Normal University Xinyang China; 2 Shanghai Chenshan Plant Science Research Center, Chinese Academy of Sciences, Shanghai Chenshan Botanical Garden, Shanghai 201602, Shanghai, China Kunming Institute of Botany, Chinese Academy of Sciences Kunming China; 3 Germplasm Bank of Wild Species, Kunming Institute of Botany, Chinese Academy of Sciences, Kunming 650201, Yunnan, China Haizhu District Experimental Primary School Guangzhou China; 4 Haizhu District Experimental Primary School, Guangzhou 510245, Guangdong, China none Weining China; 5 Weining 553100, Guizhou, China Shanghai Chenshan Botanical Garden Shanghai China

**Keywords:** *Aristolochia wardiana*, morphology, subgenus *Siphisia*, taxonomy

## Abstract

A new species of *Isotrema* was recently discovered from Guizhou, China and is here named as *I. brevilimbum*. It is most similar to *I. ovatifolium* and *I. wardianum*, but differs in the morphology of leaves and flowers. A detailed description for the new species, along with line drawings, photographs, as well as morphological comparisons with similar species, are provided. In addition, the distribution of *I. wardianum* in China is here confirmed.

## Introduction

*Isotrema* Raf. (Aristolochiaceae), previously treated as a subgenus of *Aristolochia* L., was recently reinstated as an independent genus based on molecular and morphological evidence (Zhu et al. 2019a). It can be distinguished from *Aristolochia* by the following set of characters: perianth strongly curved, gynostemium 3-lobed, anthers paired on the outer surface of each gynostemium segment, and capsule dehiscing basipetally (Do et al. 2015a; Zhu et al. 2019a). Several new species of *Isotrema* have been found and described from China and its neighbouring countries during recent years (Liu and Deng 2009; Xu et al. 2011; Yao 2012; Huang et al. 2013, 2015; Wu et al. 2013, 2015; Do et al. 2014, 2015a, 2015b, 2015c, 2015d, 2016, 2017, 2019; Huong et al. 2014; Lu and Wang 2014; Ohi-Toma et al. 2014; Zhu et al. 2015, 2016, 2017a, 2017b, 2018, 2019b, 2019c; Gong et al. 2018; Yang et al. 2018; Li et al. 2019; Peng et al. 2019; Zhou et al. 2019; Cai et al. 2020a, 2020b). Currently, a total of 106 species have been reported from *Isotrema*, most of which are distributed in eastern and southern Asia, with some species further extended to northern and central America (Zhu et al. 2019a). China accommodates ca. 66 species, among which 55 species are endemic (Huang et al. 2003; Li et al. 2019; Peng et al. 2019; Zhou et al. 2019; Zhu et al. 2019a, 2019b, 2019c, 2019d; Cai et al. 2020a, 2020b).

During our recent field explorations to southern China, an unknown species of *Isotrema* was collected. Our subsequent examination of specimens from 39 public herbaria (A, BM, BR, CDBI, CSFI, CSH, E, EMA, GXMI, HAST, HENU, HHBG, HIB, HITBC, HNWP, IBK, IBSC, K, KYO, KUN, L, LBG, LE, NAS, NTUF, P, PE, PEM, SM, SNU, SYS, TAI, TI, W, WCU, WU, WUK, XYTC, YUKU; abbreviations follow Thiers 2020) and study of related literature (Hwang 1981, 1988; Ma 1989a, 1989b; Tao 1997; Huang et al. 2003; Do et al. 2015a; Do and Nghiem 2017; Yang et al. 2018; Zhu et al. 2019a, 2019d) suggested it to be a new species. Hereafter, we describe it as *I. brevilimbum* X.X.Zhu, Jun Wang & F.Cao. Moreover, *I. wardianum* (J.S. Ma) X.X. Zhu, S. Liao & J.S. Ma was recently published (Zhu et al. 2019a) based on its basionym *A. wardiana* J.S. Ma, previously only known from Myanmar and India (Ma 1989a), which was recently collected from Medog County, Tibet, and here its distribution in China is confirmed. Measurements and morphological characters of *I. brevilimbum*, *I. ovatifolium* and *I. wardianum* were made from both dried specimens and field observations of living plants, as well as including related literature. The morphological characters of the description follow Huang et al. (2003).

## Taxonomy

### Isotrema brevilimbum

Taxon classificationPlantaeGentianalesApocynaceae

X.X.Zhu, Jun Wang & F.Cao sp. nov.

1294CE8B-7EAE-5CB4-B27D-6D6E5CF76C2D

urn:lsid:ipni.org:names:77209990-1

**Figure 1.**
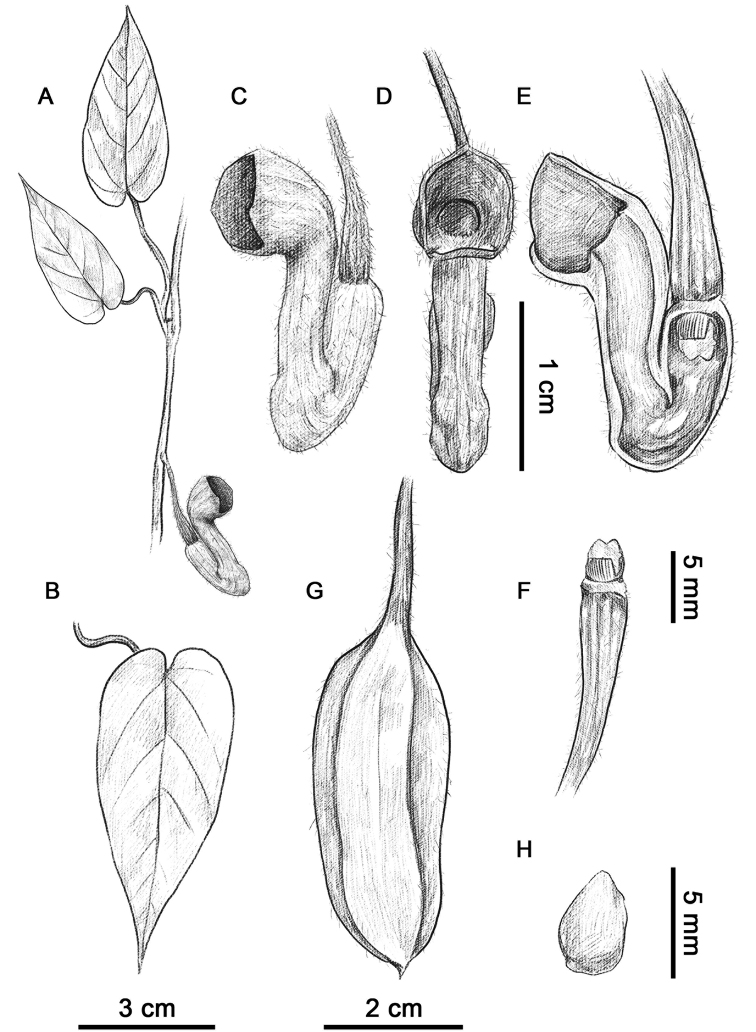
*Isotrema brevilimbum* X.X.Zhu, Jun Wang & F.Cao. **A** Branch **B** leaf **C, D** flower **E** longitudinal–section of flower (showing inside structure) **F** anthers and gynostemium **G** capsule **H** seed. Drawn by S.Z. Qiao.

**Figure 2.**
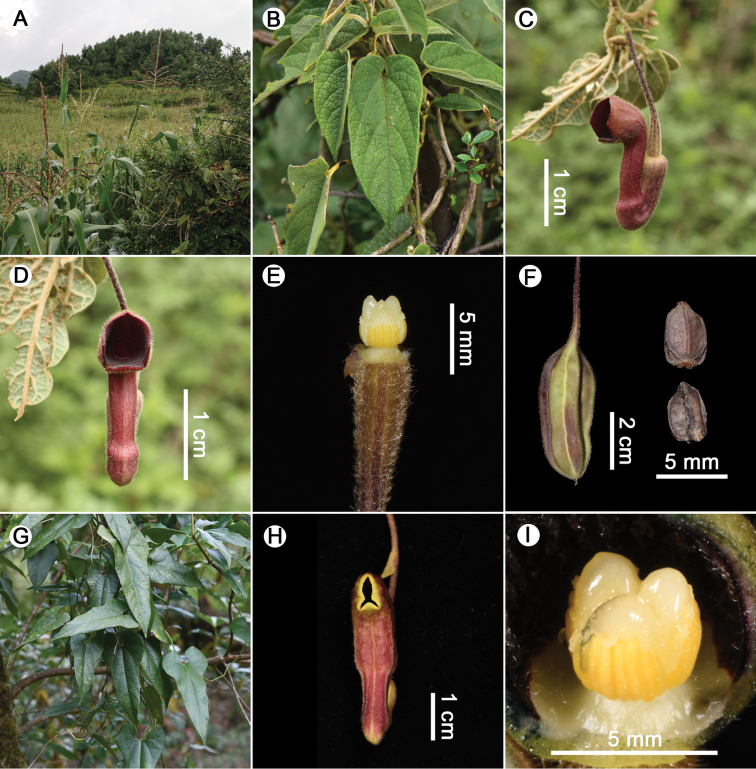
**A–F***Isotrema brevilimbum* X.X.Zhu, Jun Wang & F.Cao. **A** Habitat **B** leaves **C** lateral view of flower **D** frontal view of flower **E** anthers and gynostemium **F** capsule **G–I***I. wardianum***G** habit **H** frontal view of flower **I** anthers and gynostemium. **A** Photographed by F. Cao **B, E, F** photographed by X.X. Zhu **C, D** photographed by G. Liu **G** photographed by C. Liu **H, I** photographed by J.D. Ya.

**Figure 3.**
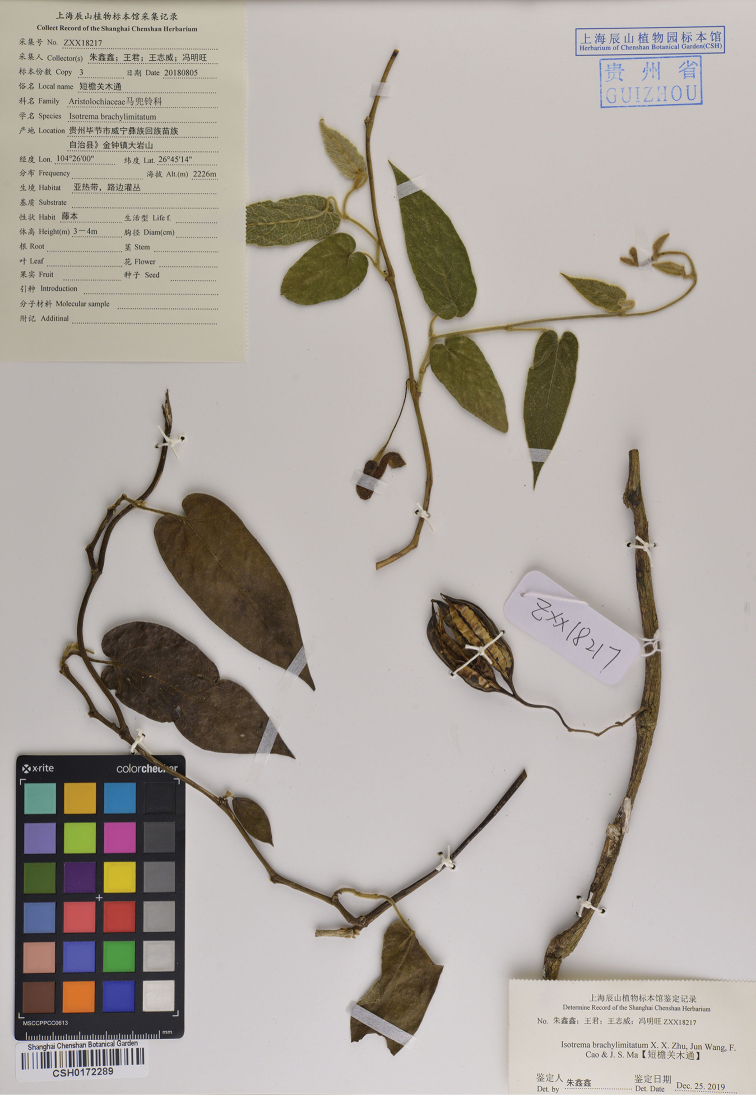
Holotype of *Isotrema brevilimbum* X.X.Zhu, Jun Wang & F.Cao (CSH–0172289).

**Figure 4.**
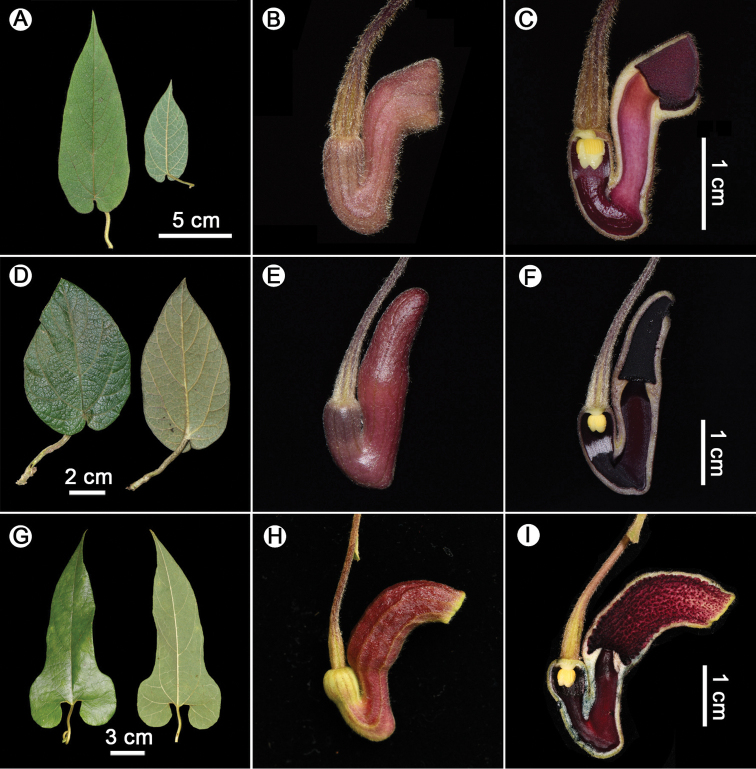
Leaves, lateral view of flowers, and longitudinal dissected flowers of *Isotrema brevilimbum* (**A–C**), *I. ovatifolium* (**D–F**), and *I. wardianum* (**G–I**). **A–F** Photographed by X.X. Zhu **G** photographed by C. Liu **H, I** photographed by J.D. Ya.

**Figure 5.**
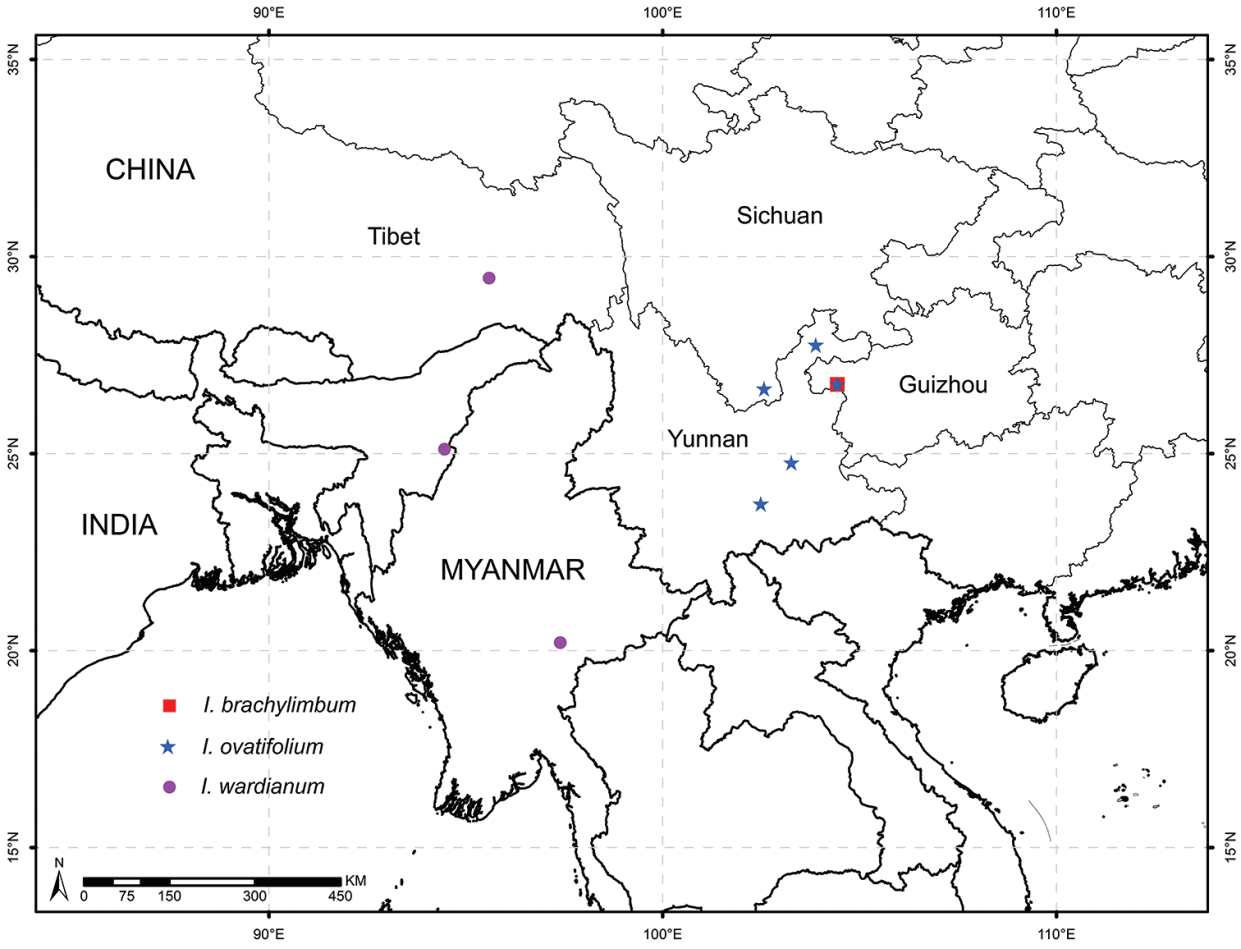
Distribution of *Isotrema brevilimbum*, *I. ovatifolium*, and *I. wardianum* based on field observation, specimens and literatures examined.

Figures 1 , 2A–F , 3 , 4A–C , 5

#### Type.

China. Guizhou: Weining County, Jinzhong Town, 2226 m alt., 5 Aug 2018, X.X. Zhu et al. ZXX18217 (holotype: CSH–0172289!; isotypes: CSH!, KUN!).

#### Diagnosis.

*Isotrema brevilimbum* is morphologically similar to *I. ovatifolium* (S.M. Hwang) X.X. Zhu, S. Liao & J.S. Ma and *I. wardianum* (J.S. Ma) X.X. Zhu, S. Liao & J.S. Ma, but differs from the former in its lamina long ovate (vs. lamina ovate in *I. ovatifolium*), perianth limb forming right angle with upper tube, length nearly equal to width, and apex dark purple and opened (vs. limb straightly extended from upper tube, length significantly longer than width, and apex dark purple and constricted in *I. ovatifolium*), differs from the latter in its lamina long ovate and abaxially densely villous (vs. lamina lanceolate and abaxially subglabrous or glabrous in *I. wardianum*), perianth limb forming right angle with upper tube, length nearly equal to width, and apex dark purple and opened (vs. limb forming obtuse angle with upper tube, length significantly longer than width, and apex light yellow and constricted in *I. wardianum*).

#### Description.

Climbing shrubs. Stems terete, densely villous when young, old branchlets glabrous. Petioles 1–4 cm long, densely villous; laminas long ovate, 5–13 × 2.5–3.5 cm, adaxially appressed villous, abaxially densely villous, base cordate, margin entire, apex acute; basal veins palmate, 2–3 pairs from base, lateral veins 4–6-paired. Flowers axillary or lateral on young stems, solitary, rarely paired. Pedicels pendulous, 1.5–3 cm long, densely villous; bracteole ovate, conduplicate, ca. 2 × 1 mm, abaxially densely villous, adaxially smooth, inserted on lower part of pedicel. Perianth tube geniculately curved, abaxially villous; basal tube ca. 1 cm long, inside dark red, upper tube ca. 1.5 cm long, inside red; limb short cylinder, length nearly equal to width, ca. 7 × 8 mm, forming right angle with upper tube, apex dark purple, opened, ca. 7 mm wide at mouth, inside dark red with densely tiny dark-purple papillae; throat subcircular, ca. 4 mm wide. Anthers 6, oblong, ca.1.5 mm long, adnate in 3 pairs to base of gynostemium, opposite to lobes. Gynostemium ca. 3 mm long, 3-lobed. Ovary terete, ca. 8 mm long, densely villous. Capsule cylindric, abaxially densely villous, ca. 4.5 × 2 cm. Seeds ovate, 4–5 × 3–3.5 mm, concave-convex.

#### Phenology.

Flowering from May to August, fruiting from July to September.

#### Etymology.

The specific epithet refers to the short cylinder perianth limb of the new species. The “brevi” means “short”, “limbum” means “limb”, so the new species is named *Isotrema brevilimbum*.

#### Common name (assigned here).

Duan Yan Guan Mu Tong (短檐关木通; Chinese name).

#### Distribution and habitat.

The new species is currently only known from Weining County of Guizhou, China. It grows by the roadside of farmland at an altitude of ca. 2200 m.

#### Preliminary conservation status.

*Isotrema brevilimbum* is known from a single population including two individuals on the roadside of farmland. The new species is assigned a preliminary status of vulnerable (VU) according to the IUCN Red List Categories (IUCN 2012). However, since very few details exist about its natural distribution, the lack of sufficient data currently does not allow a final risk evaluation and the species might be regarded as data deficient (DD). Further field surveys in western Guizhou and northeastern Yunnan are needed to gain more information on its distribution. Not only is the area not under protection as a nature reserve, but also habitat disturbance brought about by human activities, such as grazing and farming, may have a negative impact on the new species.

#### Note.

*Isotrema wardianum* was previously only known from Myanmar and India. Sun and Zhou (2002) later reported the species from China, according to a specimen collected from Medog County of Tibet (*H. Sun et al. 4935*), but without flower or fruit. Nevertheless, the species had long been neglected by taxonomic studies of Huang et al. (2003), Do et al. (2015a), and Zhu et al. (2019a, 2019d) on Chinese *Isotrema*. It was not until 2018 that we discovered a seedling of *Isotrema* sp. at the same locality as that of *H. Sun et al. 4935* and transplanted it in the nursery of the Kunming Institute of Botany. A year later, the plant grown from this seedling bloomed and enabled us to identify it as *I. wardianum* (Figs 2G–I, 4G–I) and confirm its distribution in China.

## Discussion

*Isotrema brevilimbum* is morphologically similar to *I. ovatifolium* and *I. wardianum* in the shape, size, and color of flower and the dark-purple papillae in the inner surface of perianth limb, but they can be distinguished by the morphology of lamina, the angle between perianth limb and upper tube, as well as the length and mouth of limb. Detailed morphological comparisons among the three species are summarized in Table 1 and Fig. 4.

**Table 1.** Morphological comparisons among *Isotrema brevilimbum*, *I. ovatifolium* and *I. wardianum*. These characters were based on field observation, related specimens and literatures (Hwang 1981; Ma 1989a; Huang et al. 2003).

Characters	*I. brevilimbum*	*I. ovatifolium*	*I. wardianum*
Lamina	long ovate, 5–13 × 2.5–3.5 cm, abaxially densely villous, base cordate	ovate, 5–13 × 4–8 cm, abaxially densely villous, base cordate	lanceolate, 12–16 × 3–4 cm, abaxially subglabrous or glabrous, base auriculate
Perianth limb	short cylinder, forming right angle with upper tube, length nearly equal to width, apex dark purple, opened, ca. 7 mm wide at mouth	cylinder, straightly extended from upper tube, length significantly longer than width, apex dark purple, constricted, ca. 1 mm wide at mouth	cylinder, forming obtuse angle with upper tube, length significantly longer than width, apex light yellow, constricted, ca. 3 mm wide at mouth
Perianth throat	ca. 4 mm wide	ca. 2.5 mm wide	ca. 2 mm wide
Anthers	ca. 1.5 mm long	ca. 1.5 mm long	ca. 2 mm long
Gynostemium	ca. 3 mm long	ca. 3.5 mm long	ca. 3.5 mm long
Capsule	ca. 4.5 × 2 cm	ca. 6 × 2 cm	unknown
Distribution	China (Guizhou)	China (Guizhou, Sichuan, Yunnan)	China, Myanmar, India

**Specimens of *Isotrema wardianum* examined. Myanmar.** Adung Valley, 12 Apr 1931, F. Kingdon-Ward 9398 (holotype: BM). **China. Tibet**: Medog County, 2100 m, 21 Mar 1993, H. Sun et al. 4935 (KUN); **at the same locality**, 1705 m, 27 Nov 2018, C. Liu & J.D. Ya 18CS17145 (KUN).

**Specimens of *Isotrema ovatifolium* examined. China. Guizhou**: Weining County, Jinzhong Town, 2232 m, 5 Aug 2018, Zhu et al. ZXX18218 (CSH, KUN); **Sichuan**: Huidong County, 2520 m, 27 Jun 1959, S.K. Wu 1584 (type: SM).

## Supplementary Material

XML Treatment for Isotrema brevilimbum
